# Methylated arsenic metabolites bind to PML protein but do not induce cellular differentiation and PML-RARα protein degradation

**DOI:** 10.18632/oncotarget.4662

**Published:** 2015-07-15

**Authors:** Qian Qian Wang, Xin Yi Zhou, Yan Fang Zhang, Na Bu, Jin Zhou, Feng Lin Cao, Hua Naranmandura

**Affiliations:** ^1^ Department of Toxicology, School of Medicine and Public Health, Zhejiang University, Hangzhou 310058, China; ^2^ College of Pharmaceutical Sciences, Zhejiang University, Hangzhou 310058, China; ^3^ Department of Hematology and Oncology, The First Clinical College of Harbin Medical University, Harbin 150086, China

**Keywords:** acute promyelocytic leukemia, arsenic trioxide, trivalent arsenicals, arsenic binding proteins, monomethylarsonous acid

## Abstract

Arsenic trioxide (As_2_O_3_) is one of the most effective therapeutic agents used for patients with acute promyelocytic leukemia (APL). The probable explanation for As_2_O_3_-induced cell differentiation is the direct targeting of PML-RARα oncoprotein by As_2_O_3_, which results in initiation of PML-RARa degradation. However, after injection, As_2_O_3_ is rapidly methylated in body to different intermediate metabolites such as trivalent monomethylarsonous acid (MMA^III^) and dimethylarsinous acid (DMA^III^), therefore, it remains unknown that which arsenic specie is actually responsible for the therapeutic effects against APL. Here we have shown the role of As_2_O_3_ (as iAs^III^) and its intermediate metabolites (i.e., MMA^III^/DMA^III^) in NB4 cells. Inorganic iAs^III^ predominantly showed induction of cell differentiation, while MMA^III^ and DMA^III^ specifically showed to induce mitochondria and endoplasmic reticulum-mediated apoptosis, respectively. On the other hand, in contrast to iAs^III^, MMA^III^ showed stronger binding affinity for ring domain of PML recombinant protein, however, could not induce PML protein SUMOylation and ubiquitin/proteasome degradation. In summary, our results suggest that the binding of arsenicals to the ring domain of PML proteins is not associated with the degradation of PML-RARa fusion protein. Moreover, methylated arsenicals can efficiently lead to cellular apoptosis, however, they are incapable of inducing NB4 cell differentiation.

## INTRODUCTION

Arsenic trioxide (As_2_O_3_) has been accepted as a standard treatment for the patients with acute promyelocytic leukemia (APL) in China [1], which has also been applied for the treatment of other forms of cancers [2–3]. Although, As_2_O_3_ has been studied *in vivo* and *in vitro* for many years, however, there is a little information regarding the anticancer effect of the intermediate metabolites of As_2_O_3_, namely, monomethylarsonous acid (MMA^III^) and dimethylarsinous acid (DMA^III^) in APL patients receiving As_2_O_3_ treatment.

Generally speaking, liver is the major site for arsenic methylation, where As_2_O_3_ is metabolically transformed into trivalent mono- and di-methylated metabolites (i.e., MMA^III^ and DMA^III^) by arsenicmethyltransferase (AS3MT) [4]. Finally, it is excreted into urine mostly in the form of pentavalent methylated metabolites such as monomethylarsonic acid (MMA^V^) and dimethylarsinic acid (DMA^V^) [5–6]. In fact, methylated pentavalent arsenic species; MMA^V^ and DMA^V^ could frequently be found in the bloodstream and urine of APL patients after injection of As_2_O_3_ [7–8]. Wang et al. (2004) has reported that the highly toxic trivalent arsenic metabolite, MMA^III^ was found in the urine of APL patients receiving As_2_O_3_ injection [8]. Likewise, it was also found in human saliva and urine following exposure to iAs^III^ in Inner Mongolia [9].

Toxicological studies have recently indicated that trivalent arsenic intermediate metabolites (i.e., MMA^III^ and DMA^III^) are more toxic as compared to their precursor; arsenite (iAs^III^) [10], and these trivalent arsenicals have also shown to display a much higher degree of cytotoxicity than the corresponding pentavalent species. However, little is known about the molecular role of these active trivalent intermediate arsenicals in the clinical remission of APL patients. Chen et al. (2003) has reported that methylated MMA^III^ may contribute to arsenic-induced apoptosis in leukemia and lymphoma cells [11], however, no detailed mechanism has been investigated so far. Based on such observations, it can be suggested that the trivalent methylated arsenicals may contribute to the therapeutic effects in APL.

APL is characterized by a reciprocal translocation between chromosomes 15 and 17, t(15;17), expressing the fusion of promyelocytic leukemia (*PML*) gene to the retinoic acid receptor α (*RARα*) gene resulting in the production of a PML-RARα fusion protein [12], this fusion protein may block the differentiation of hematopoietic progenitor cells [13–14]. On the other hand, PML-RARα regulated adapter molecule-1 (PRAM-1), an adaptor protein which is expressed and regulated during normal myelopoiesis is found to be down-regulated by PML-RARα fusion protein in NB4 cells, suggesting important contribution of this protein in signaling pathway involved in differentiation of leukemia promyelocytes [15].

Successful clinical remission in APL patients has been obtained with all-trans retinoic acid (ATRA) and As_2_O_3_ treatment [16–17]. Zhang et al. has reported that As_2_O_3_ may directly bind to cysteine residues in the RING finger-B Box-Coiled Coil (RBCC) domain of PML-RARα fusion protein, which results in enhanced SUMOylation and degradation of PML-RARα fusion protein, promoting cell differentiation leading to clinical remission [18]. Moreover, binding of trivalent arsenicals to the zinc finger domains of DNA repair proteins and cysteine rich metallothionein (MT) has extensively been studied recently [19]. Although, at normal physiological conditions, trivalent arsenicals have shown to provoke zinc release from the zinc finger domain of the xeroderma pigmentosum group A protein (XPA), the methylated metabolites; MMA^III^ and DMA^III^ were found to have stronger binding affinity to zinc finger proteins as compared to iAs^III^ [20]. In fact, the studies that have shown binding of iAs^III^ to zinc finger peptides were mostly performed using apo-Zn finger proteins [18–19], thus, more studies are needed to evaluate that whether iAs^III^ can also bind to Zn finger peptides or proteins (i.e., with Zn^2+^) even under normal physiological conditions.

Scientists have mainly focused on the effect of As_2_O_3_ on APL [21–22], showing a minimal concern to reveal the effects of the intermediates MMA^III^ and DMA^III^ on treatment of APL patients. Based on the above observations, we are interested in whether the degradation of PML-RARα fusion is related to the binding of As_2_O_3_ and its intermediate metabolites. Thereby, in the current investigation we opt to determine the effect of the three arsenicals, including iAs^III^ (represent the As_2_O_3_) and its two intermediate metabolites (i.e., MMA^III^/DMA^III^) on induction of cellular differentiation and apoptosis. In addition, we further compared the binding affinity of iAs^III^ and its intermediate metabolites to the recombinant PML-zinc finger protein to reveal the probable association between the arsenic binding to protein and PML degradation. Interestingly, our present work demonstrated that iAs^III^ predominantly activates cells differentiation, while its intermediate MMA^III^ and DMA^III^ specifically induce apoptosis. Moreover, it was found that binding of arsenic intermediate metabolites to PML-zinc finger protein do not induce the PML-protein degradation.

## RESULTS

### The proliferation of NB4 cells after exposure to iAs^III^ and MMA^III^ and DMA^III^

**Figure 1:**
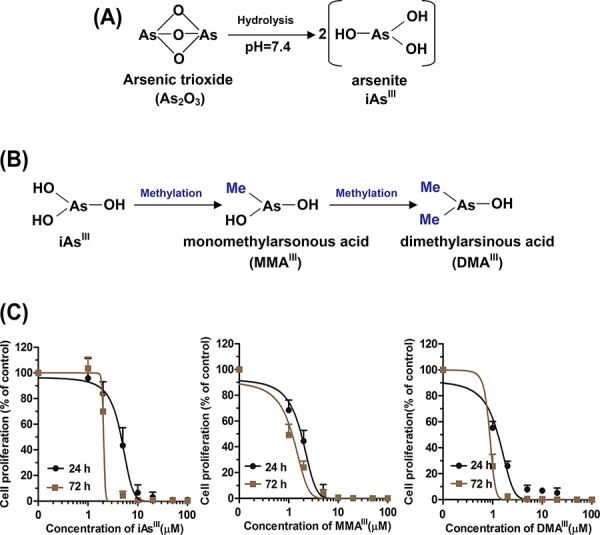
Proposed metabolic pathway for arsenic trioxide *in vivo* and proliferation of NB4 cells after exposure to arsenicals Arsenic trioxide is commonly hydrolyzed into arsenite (iAs^III^) at the physiological conditions **A.** iAs^III^ can immediately be methylated to MMA^III^ and DMA^III^ in body **B.** Cells were exposed to various concentrations of arsenicals for 24 and 72 h, and then viability was determined by MTT assay **C.** Data are expressed as mean values ± standard deviation (*n* = 4).

As_2_O_3_ in body is commonly present in its hydrolyzed form (e.g., As_2_O_3_ → iAs^III^) (Fig. 1A), thereby, in current study, iAs^III^ will represent As_2_O_3_. In humans, iAs^III^ has found to be rapidly methylated to trivalent mono- and di- methylated metabolites (i.e., MMA^III^ and DMA^III^) by arsenic methyltransferse (AS3MT) in liver (Fig. 1B), however, little is known about the therapeutic effects of intermediate metabolites of iAs^III^ on APL patients receiving As_2_O_3_ treatment. Here, we determined the effects of iAs^III^, MMA^III^ and DMA^III^ on the NB4 cell proliferation, where MMA^III^ and DMA^III^ were found to be more cytotoxic to NB4 cells as compared to iAs^III^ (Fig. 1C). In addition, we also determined the cell viability by trypan blue exclusion assay, the data of cell viability were consistent with MTT assay (data not shown). On the other hand, according to the dosage of As_2_O_3_ (0.15 mgAs/kg) in clinical treatment [1, 16–18] as well as concentration of arsenic species in blood samples [23], we selected 1 μM dose of arsenic compounds for our subsequent experiments.

### Effects of arsenicals on NB4 cells differentiation or on PML-RARα fusion protein degradation as well as PML nuclear bodies (PML-NBs) formations

**Figure 2:**
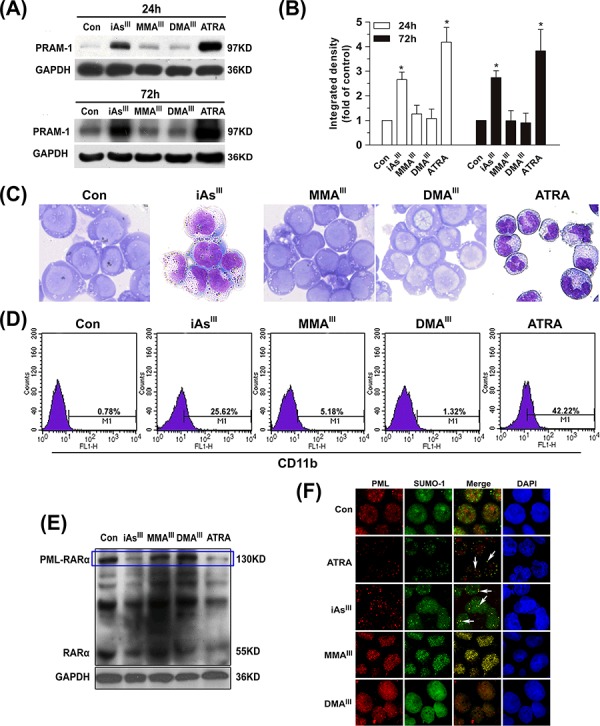
Effects of arsenicals on differentiation of NB4 cells and PML-RARα fusion protein degradation NB4 cells were exposed to 1 μM of arsenicals for 24 and 72 h to determine the changes in PRAM-1 protein **A.** and quantified by Image J public domain software **B.** Additionally, NB4 cells were stained with Wright-Giemsa **C.** and the expression of CD11b in NB4 cells were determined by flow cytometry **D.** Degradation of PML-RARα fusion protein in NB4 cells were determined by western blot with RARα antibody **E.** For immunofluorescence, NB4 cells were double-labeled with PML (red) and SUMO-1 (green) after exposure to 1 μM arsenicals for 12 h. Blue fluorescence indicates cell nucleus. Cells were imaged with a laser scanning confocal microscope **F.** ATRA was used as positive control in the present study. Asterisks indicate a significant difference from the untreated control group at *p < 0.05. Arrow indicates the PML-NBs.

In order to better understand the role of three arsenic species in NB4 cells, cellular differentiation and PML-RARα fusion protein degradation were determined at 24 or 72 h following exposure to arsenicals (Fig. 2).

The expression of PRAM-1 was initially found to be very low in NB4 cells, however, its expression markedly increased as early as 6 h after exposure to iAs^III^ (Fig. S1A). Moreover, expression of PRAM-1 was observed to be further increased after 24/72 h of exposure to iAs^III^ and/or ATRA. However, there were no appreciable changes observed after exposure to either MMA^III^ or DMA^III^ (Fig. 2A–2B). Moreover, the results of Wright-Giemsa stain and CD11b clearly showed cellular differentiation induced by both iAs^III^ and ATRA, however, no cellular differentiation in NB4 cells was observed after exposure to MMA^III^ or DMA^III^ at 1 μM (Fig. 2C, 2D). Likewise, iAs^III^ (or ATRA)-treated cells displayed typical nuclear morphology of differentiation (i.e., polylobular nuclei) as compared to control, while MMA^III^ and DMA^III^-treated cells have shown apoptotic chromosome condensation and fragmented nuclei (Fig. S1B).

PML-RARα fusion protein degradation in NB4 cells was observed to occur as early as 6 h after exposure to iAs^III^ and ATRA (Fig. S1C). However, MMA^III^ or DMA^III^ could not degrade PML-RARα fusion protein at any time point (Fig. 2E), suggesting that these metabolites may not have any effect on NB4 cell differentiation and PML-RARα protein degradation. In order to confirm whether massive apoptosis might inhibit their (i.e., MMA^III^ and DMA^III^) potential ability of promoting cellular differentiation, we further used lower doses of methylated arsenicals (e.g., 0.1 and 0.5 μM) to determine NB4 cell differentiation (Fig. S1). Our results clearly found that although both methylated MMA^III^ and DMA^III^ did not induce apoptosis significantly at lower doses, (Fig. S1E), they were also unable to induce cell differentiation (Fig. 1D).

It is known that iAs^III^ and ATRA are able to redirect wild-type PML from aberrant subnuclear distribution in APL cells to its normal localization [24]. Thereby, trying to understand whether the methylated arsenic species could induce PML-NBs formation/SUMOylation, the co-localization of PML with small ubiquitin-like modifier 1 (SUMO-1) in NB4 cells were examined after exposure to three arsenic species. We found that iAs^III^ and ATRA remarkably induced a characteristic redistribution of PML together with SUMO-1 to form the nuclear speckled structures as compared to control, while the treatment with either MMA^III^ or DMA^III^ showed no effect (Fig. 2F), suggesting that the methylated trivalent arsenic species can not trigger the relocalization of the PML-NBs in NB4 cells.

### MMA^III^ or DMA^III^ have strong effect on induction of apoptosis in NB4 cells

**Figure 3:**
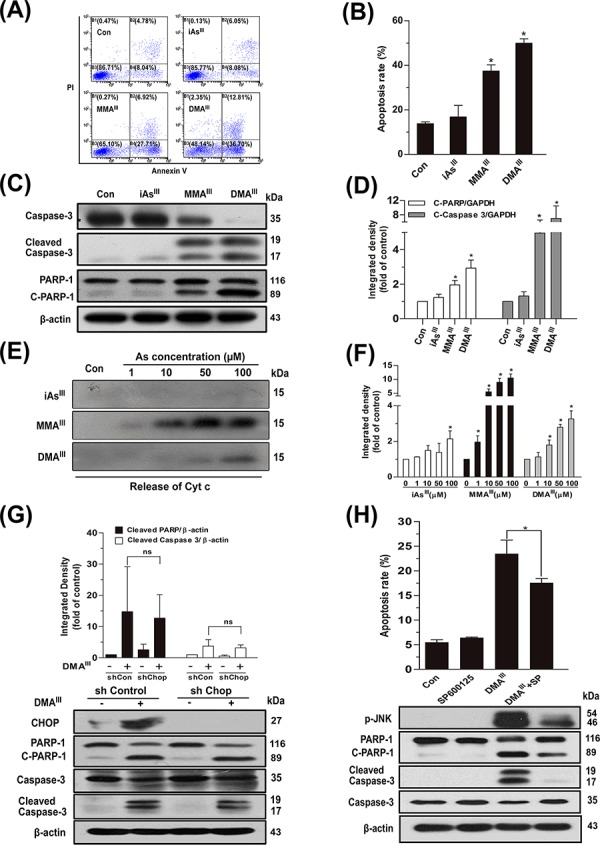
Effect of arsenicals on induction of apoptosis in NB4 cells or release of Cyt c from intact mitochondria Apoptosis in NB4 cells was determined by flow cytometry following exposure to 1 μM of arsenicals for 24 h **A-B.** Apoptosis-related proteins were determined by western blot **C.** and quantified by Image J public domain software **D.** Isolated pure rat liver mitochondria (200 μg protein/ml) were incubated with three arsenicals with indicated concentration for 30 min, and the release of Cyt c in supernatant was determined by western blot **E, F.** Apoptosis was determined by knockdown of CHOP by shRNA **G.** or inhibition of p-JNK using its specific inhibitor SP600125 **H.** in NB4 cells following exposure to DMA^III^. n.s. (not significant).

Based on the above results, we hypothesized that MMA^III^ and DMA^III^ may contribute to induce apoptosis in NB4 cells. As anticipated, we found that MMA^III^ and DMA^III^ (1 μM) significantly induced apoptosis in NB4 cells, while no appreciable effect was observed after exposure to iAs^III^ at 1 μM (Fig. 3A–3D), where the toxicity of DMA^III^ was observed to be greater than MMA^III^.

On the other hand, apoptosis related proteins such as cleaved caspase-3 and poly (ADP-ribose) polymerase (PARP) were clearly observed at early time following exposure to MMA^III^ and DMA^III^, which were found to increase in a time- and dose-dependent manner (Fig. S2). Similarly, proapoptotic protein, Bcl-2-associated X protein (Bax) and/or anti-apoptotic protein, B-cell lymphoma 2 (Bcl-2), both were found to significantly decrease in cytoplasm and mitochondria as early as 6 to 12 h after exposure to MMA^III^ and DMA^III^, and continuously decreased till 24 h (Fig. S3A/C). Especially, Cyt c was markedly found to be released from mitochondria in the cytoplasm of NB4 cells, and increased with exposure time of MMA^III^ and DMA^III^ as compared to iAs^III^ (Fig. S3B/D). Likewise, the mitochondrial membrane potential (ΔΨ_m_) was also significantly reduced by MMA^III^ and DMA^III^, suggesting that these trivalent intermediates indeed contributed in the induction of apoptotic cell death (Fig. S3E).

To reveal the mechanism underlying the release of Cyt c, pure rat liver mitochondria were incubated with three arsenic species for 30 min. Surprisingly, iAs^III^ was unable to release Cyt c from intact mitochondria (even at 100 μM), while methylated MMA^III^ showed to have potential effect on the release of Cyt c from intact mitochondria, which was observed to be much stronger than DMA^III^ (Fig. 3E–3F). Similarly, MMA^III^ can induce opening of mPTP even at low concentrations; however, DMA^III^ only showed effect at high concentration, while no effects were observed with iAs^III^ (data not shown). However, we found that DMA^III^–induced increase in cytoplasmic Cyt c (Fig. S3D), was not consistent with the data of intact mitochondria (Fig. 3F). Thereby, these apparently contradictory results call into question the signaling pathway that could be involved in DMA^III^–induced apoptosis.

We hypothesized that DMA^III^ may target endoplasmic reticulum and may show its effect via ER-related pathways. Interestingly, we found that the phosphorylated protein kinase-like endoplasmic reticulum kinase (PERK) was significantly activated in NB4 cells after exposure to DMA^III^ as compared to the other two arsenic species; iAs^III^ and MMA^III^ (Fig. S4A), suggesting DMA^III^ as a potent inducer of ER stress. Similarly, a significant increase in the expressions of p-PERK and α submits of eukaryotic translation initiation factor-2 (elF2α), or downstream activating transcription factor 4 (ATF4) and C/EBP homologous protein (CHOP) were observed following exposure to DMA^III^ (Fig. S4B). Likewise, DMA^III^ was also capable of inducing JUN N-terminal kinase (JNK) phosphorylation in NB4 cells and triggering JNK dependent ER-stress-induced apoptosis (Fig. S4C–S4D). Moreover, after the knockdown of CHOP, no appreciable difference was observed in apoptosis related proteins such as PARP and caspase-3 between the shCHOP and shControl group (Fig. 3G), however, inhibition of p-JNK significantly prevented cell death in NB4 cells after exposure to DMA^III^ (Fig. 3H).

### Determination of formation of nuclear bodies in PML and PML-RARα-transfected cells after exposure to arsenicals

As_2_O_3_ can induce formation of PML-NBs, SUMOylation of PML-RARα (or PML) protein in APL cells [25]. In the current study, we found that MMA^III^ and DMA^III^ could not induce relocalization of PML-NBs, SUMOylation and PML-RARα degradation in NB4 cells (Fig. 2). Thus, HEK293T and HeLa cells were transiently transfected with *PML* or *PML-RARα* genes to confirm the effect of the three arsenicals on PML/PML-RARα protein degradation, relocalization of PML-NBs, SUMOylation. We found that the *PML* and *PML-RARα* successfully overexpressed in both cell lines; HEK293T and HeLa cells (Fig. S5A–S5B).

**Figure 4:**
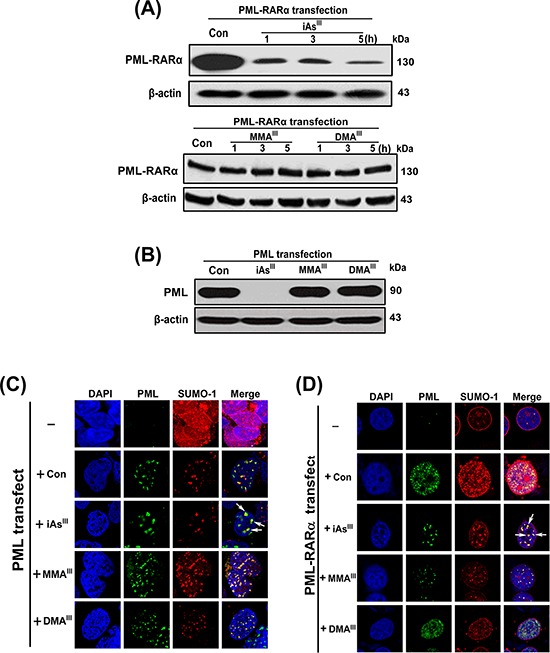
Examination of PML-NBs formation or PML and PML-RARα proteins degradation in HEK293T and HeLa cells after exposure to arsenicals Degradation of PML-RARα fusion protein was examined in *PML-RARα*-transfected HeLa cells following exposure to 4 μM of arsenicals at indicated time points **A.** Likewise, degradation of PML protein was determined in *PML*-HEK293T cells following exposure to 4 μM of arsenicals for 6 h **B.** Formation of PML-NBs in *PML* or *PML-RARα* overexpressed HEK293T **C.** or HeLa cells **D.** were determined after exposure to arsenicals for 6 h. Cells were double-labeled with PML (green) and SUMO-1 (red). Blue fluorescence indicates cell nucleus. Arrow indicates the PML-NBs.

Furthermore, methylated MMA^III^ and DMA^III^ were found unable to degrade the PML or PML-RARα proteins in HEK293T and HeLa cells, however, iAs^III^ showed to induce complete degradation of these proteins (Fig. 4A–4B). These results were found be consistent with our above data obtained using NB4 cells (Fig. 2E). Similarly, re-localization of PML-NBs in *PML*-HEK293T cells or *PML-RARα*-HeLa cells was examined after exposure to the three arsenicals. The green fluorescence indicates PML, while the red fluorescence indicates SUMO-1. As anticipated, only iAs^III^ induced a characteristic redistribution of PML together with SUMO-1 to form the nuclear speckled structures as compared to control (Fig. 4C–4D), while the NBs are dispersed as microspeckles in *PML*-HEK293T cells or *PML-RARα*-HeLa following exposure to methylated arsenicals (Fig. 4C–4D), suggesting that MMA^III^ and DMA^III^ have no effect on PML protein degradation and PML-NBs formation.

### Binding of arsenic species with PML-Zinc finger protein and other proteins in NB4 cells

It has been indicated that iAs^III^ directly binds to cysteine residues of zinc fingers located within the RBCC domain of PML-RARα resulting in degradation of PML-RARα fusion protein. Thus, to address the binding affinity of the three arsenic species to the cellular proteins, we used a gel filtration GS-220 column and determined the arsenic-binding proteins and unbound arsenicals in NB4 cells after exposure to 1 μM of arsenicals for 24 h.

**Figure 5:**
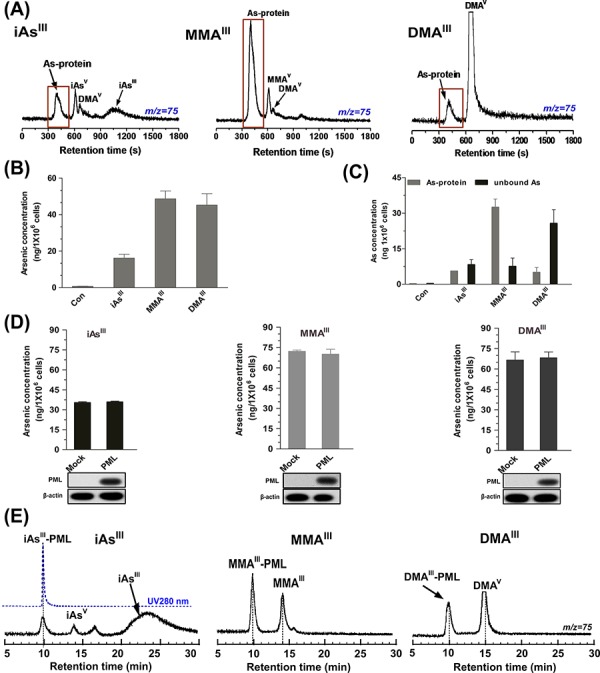
Analysis of arsenic distribution and concentration in NB4 cells after exposure to three arsenic compounds NB4 cells were treated with 1 μM of arsenicals for 24 h. Arsenic species in supernatant were determined by ICP-MS on GS-220 column with 50 mM ammonium acetate (pH6.5) at the flow rate of 0.6 mL/min **A.** Arsenic accumulation in NB4 cells **B.** or concentration of arsenic-binding proteins and free arsenic (unbound) **C.** were measure by ICP-MS after ashing with HNO_3_ and H_2_O_2_. **D.** Arsenic accumulation in Mock or *PML* transfected HEK293T cells after exposure to 2 μM arsenicals for 6 h was measure by ICP-MS after washing with HNO_3_ and H_2_O_2_. **E.** Binding of three arsenic species to recombinant PML-R protein (25 μg/mL) was determined by ICP-MS on GS-220 column with 50 mM ammonium acetate (pH7.0) at the flow rate of 0.8 mL/min. Arsenic was monitored at m/z 75.

We detected few arsenic-binding proteins along with a large amount of free iAs^III^ (unbound-form) in NB4 cells after exposure to iAs^III^ (Fig. 5A). However, in the MMA^III^ treated cells, most of the arsenic was detected in protein-binding form and no free MMA^III^ was observed. Moreover, only a small amount of arsenic was found to bind to proteins, and most of the arsenic was detected as DMA^V^ following exposure to DMA^III^ (Fig. 5A). Thus, these data indicated that MMA^III^ has much stronger binding affinity to SH-group of proteins as compared to iAs^III^ or DMA^III^. Additionally, MMA^III^ and DMA^III^ were more efficiently taken up by NB4 cells than the precursor iAs^III^ (Fig. 5B), where, most of MMA^III^ was found to bind to proteins in whole cell extract (Fig. 5C).

We were further interested in detecting that whether overexpression of *PML* in HEK293T cells can also increase arsenic accumulations, vector or *PML* transfected HEK293T cells were exposed to 2 μM of three arsenicals for 6 h. Interestingly, no significant differences were noted in the results between the *PML* and Mock-transfected HEK293T cells after exposure to the three arsenicals (Fig. 5D). Furthermore, we evaluated the binding affinity of the three arsenicals to the zinc fingers in the RBCC domain of PML-RARα fusion protein. For this, the recombinant zinc finger protein PML-Ring (containing Zn^2+^) was incubated with the three arsenicals for 30 min in Tris-HNO_3_ buffer (pH7.4). Surprisingly, inorganic iAs^III^ was found unable to bind to PML-R at physiological condition (pH7.4), while MMA^III^ exhibited high binding affinity for recombinant PML-R (Fig. 5E). Although DMA^III^ was capable of binding to PML-R, it has exhibited less efficiently as compared to MMA^III^. Here, we concluded that although intermediate metabolites have a high binding affinity for zinc finger proteins, they could not degrade PML or PML-RARα proteins in NB4 cells or *PML*-transfected HEK293T cells, suggesting that binding to zinc finger proteins may not be a requirement for PML-RARα degradation.

### Determination of PML proteins SUMOylation and ubiquitination after exposure to arsenicals

**Figure 6:**
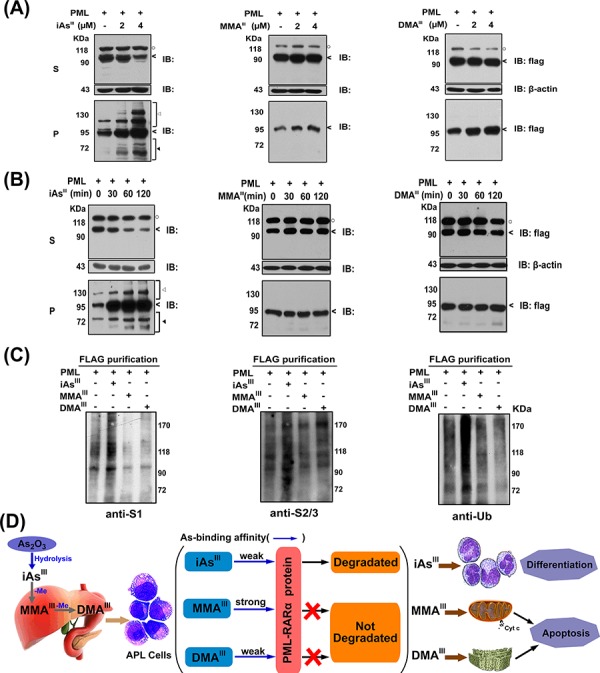
Changes in PML protein in soluble (S) and insoluble fractions (P) of *PML*-HEK293T cells by exposure to arsenicals in time and dose-dependent manners *PML* overexpressed-HEK293T cells were exposed to 2 μM of arsenicals in time **A.** or dose-dependent **B.** manners. Arsenic-treated cells were lysed in RIPA buffer, centrifuged into supernatant (S) and pellet (P) fractions, and then determine the PML proteins (<), modified PML-proteins (◄) and degraded PML-protein (◄) by western blot analysis. (○) indicates a non-specific protein in supernatant. **C.** PML protein was immunoprecipitated from extract of *PML* overexpressed-HEK293T cells by anti-FLAG-M2 beads after exposure to 2 μM three arsenic species for 2 h. SUMO-1, 2/3 and ubiquitin antibodies were used to determine the modification of PML proteins (anti-S1: SUMO-1; anti-S2/3: SUMO-2/3; anti-Ub: ubiquitin). **D.** Proposed mechanism underlying the arsenic trioxide and its methylated metabolites-induced cell differentiation and apoptosis in NB4 cells.

In order to confirm the effect of three arsenicals on modification of PML proteins, *PML*-HEK293T cells were exposed to three arsenicals in a concentration and time dependent manner. Cells were lysed with RIPA buffer, and then separated into soluble (Supernatant) and insoluble fractions (Pellet). Interestingly, iAs^III^ was observed to induce a shift of PML protein from supernatants to insoluble fractions in a concentration-dependent manner, while no changes were observed following exposure to MMA^III^ and DMA^III^ (Fig. 6A). Meanwhile, this shift of PML was found to occur as early as 30 min by iAs^III^ and then increased with exposure time. However, the result of methylated metabolites was found to be in contrast to this (Fig. 6B/Fig. S5C) i.e., having no effect even with the increase in the exposure time. These data indicated that only iAs^III^ can induce PML modification.

Based on above observation, we hypothesize that methylated arsenic species probably have no effect on induction of SUMOylation/ubiquitination of PML proteins. Thus, SUMOylation/ubiquitination of PML proteins was determined in *PML*-HEK293T cells by immunoprecipitation using specific antibodies after exposure to three arsenic species (Fig. 6C). As anticipated, PML proteins were modified to SUMO-1, SUMO-2/3, and ubiquitin conjugations after exposure to iAs^III^, but no changes were observed following exposure to both MMA^III^ and DMA^III^, suggesting that methylated arsenic species could not induce PML proteins SUMOylation as well as ubiquitination.

## DISCUSSION

In the present study, we clearly demonstrated that iAs^III^ and its intermediate metabolites exert different roles in NB4 cells; especially MMA^III^ and DMA^III^ have shown to have no effect on NB4 cell differentiation, however, they showed to have strong effect on inducing NB4 cell apoptosis compared with iAs^III^. This clearly suggests that the formation of these methylated metabolites may probably contribute to the antileukemic effect in APL patient after As_2_O_3_ treatment through other pathways. In fact, liver is considered to be the major organ for arsenic methylation, where As_2_O_3_ is rapidly methylated to different intermediates such as trivalent mono- and dimethylated arsenic species by AS3MT, and then redistributed to body fluids and other organs [6]. However, Khaleghian et al. (2014) has recently reported that NB4 cells are capable of metabolizing As_2_O_3_ into methylated metabolites, suggesting that the production of these methylated metabolites may contribute to the therapeutic effect of As_2_O_3_ in APL treatment [26].

In fact, DMA^III^ showed higher cytotoxic activity against NB4 cells than MMA^III^ (Fig. 3), and induced abundance of Cyt c release into the cytosolic (Fig. S3), suggesting that DMA^III^ may also target cellular mitochondria for the induction of apoptosis. Contrary to our hypothesis, MMA^III^ was shown to have more potent ability to release Cyt c from intact mitochondria, while only a small amount of Cyt c was released by DMA^III^ at high dose (Fig. 3E), indicating that MMA^III^ can directly attack mitochondria. Thus, we hypothesize that the DMA^III^-induced release of Cyt c in NB4 cells could have probably occurred through other signaling pathways.

Expectedly, ER-stress can be specifically induced by DMA^III^, however, little changes were observed following exposure to iAs^III^ and MMA^III^ (Fig. S4A–S4B). Additionally, activation of JNK is a mediator of ER stress-induced apoptosis [27], and ER stress is majorly responsible for JNK activation [28]. Here, DMA^III^ significantly induced apoptosis signal regulating kinase 1 (ASK1)-JNK activation (Fig. S4C–S4D), and inhibition of p-JNK could prevent DMA^III^-induced apoptosis (Fig. 3H). These results suggest that the activation of ASK1-JNK is involved in the induction of apoptosis via ER stress. Our results demonstrated that MMA^III^ directly attacks the mitochondria to induce apoptosis, while DMA^III^–induced apoptosis is through induction of ER stress (Fig. S6).

Trivalent arsenicals are known to be highly reactive towards the free-cysteine residues in proteins [29]. Especially, MMA^III^ have shown strong ability to release zinc ion from the zinc finger protein, and binds to the protein [20]. In NB4 cells, MMA^III^ showed stronger binding affinity to proteins as compared to iAs^III^ and DMA^III^ (Fig. 5A). Correspondingly, MMA^III^ is able to release Zn^2+^ ions and bind to Ring domain of PML-R recombinant protein at physical condition (pH7.4), but DMA^III^ exhibited less efficiently as compared to MMA^III^ (Fig. 5E). Furthermore, little amount of iAs^III^ was also observed to bind to PML-R protein. This is probably because a small amount of zinc might be released from the PML-R during the purification process providing free cysteine residues for iAs^III^ binding. In addition, our results are consistent with previous studies; proposing that iAs^III^ could not bind to zinc finger protein XPAzf (containing Zn^2+^) in ammonium acetate buffer (pH7.4) as determined by ESI-MS, while MMA^III^ exhibits potent binding affinity for XPAzf [20]. Collectively, it seems that at physiological condition, binding of iAs^III^ to Zinc finger proteins (except to the apo-Zinc finger protein; without Zn^2+^ ions) is difficult however, the methylated arsenic compounds demonstrate to have strong binding affinity for PML or other thio-containing proteins.

Notably, although MMA^III^ was able to bind to ring domain of PML protein, the methylated arsenicals could not induce NB4 cell differentiation or PML-RARα protein degradation (Fig. 2). Similarly, we also further confirmed that PML proteins could not be degraded by exposure to both MMA^III^ and DMA^III^ in *PML* or *PML-RARα* overexpressed HEK293T or HeLa cells, however, these proteins were completely degraded by iAs^III^, which was found to be consistent with previous reports [30–31]. Additionally, there was no re-localization of PML-NBs found in HEK293T or HeLa cells after exposure to the two methylated arsenic species (Fig. 4C–4D), indicating that the arsenic binding to PML protein actually does not correlate with PML protein degradation. Moreover, our results of immunoprecipitation have clearly shown that the two methylated arsenic species are incapable of inducing PML protein SUMOylation and ubiquitination (Fig. 6C), which clearly indicates that iAs^III^ once methylate to organic compounds, it will lose its ability to induce cell differentiation or PML protein degradation.

In summary, we suggest that the three different arsenic species exert different roles in NB4 cells; where, iAs^III^ can predominantly induce cellular differentiation, while the two intermediate metabolites; MMA^III^ and DMA^III^ can induce cellular apoptosis as schematically drawn in Figure 6D. However, further studies are required to confirm whether the formation of the arsenic intermediate metabolites (i.e., induction of apoptosis) in APL patients may help increase the therapeutic efficacy of As_2_O_3_ treatment.

## MATERIALS AND METHODS

### Reagents

All reagents were of analytical grade. Milli-Q water (Millipore) was used throughout the experiment. Trizma^®^ HCl and Trizma^®^ Base were purchased from Sigma (St. Louis, MO, USA). Nitric acid, ammonium acetate, acetic acid, 28% ammonia solution, L-cysteine, sodium arsenite (iAs^III^), sodium arsenate (iAs^V^), and dimethylarsinic acid [(CH3)2AsO(OH)] (DMA^V^) were purchased from Wako Pure Chemical Industries, Ltd. (Osaka, Japan). Monomethylarsonic acid (MMA^V^) was obtained from Tri Chemicals (Yamanashi, Japan). The arsenic standard solution (1, 000 μg/mL) for ICP-MS was purchased from SPEX CentiPrep (Metuchen, NJ, USA). Stock solutions of all arsenic compounds (10 mmol/L) were prepared from the respective standard compounds. All stock solutions were stored in the dark at 4°C. Diluted standard solutions for analysis were prepared daily prior to use.

### Cell culture

NB4 cells were kindly provided by Dr. Qiaojun He (Zhejiang University, China) in September 2012 and no authentication was done by the author. HEK293T and HeLa cells were purchased from Cell Bank of China Science in April 2013. Following receipt, cells were grown and frozen as a seed stock as they were available. Cells were passaged for a maximum of 3 months, after which new seed stocks were thawed. Two cell lines were authenticated using DNA fingerprinting (variable number of tandem repeats), confirming that no cross-contamination occurred during this study. Two cell lines were tested for mycoplasma contamination at least two times per year.

Cells (1.0 × 10^6^) were cultured in T25 flask, and were maintained in logarithmic growth phase using RPMI-1640 or DMEM medium supplemented with 10% fetal bovine serum (FBS), 100 U/mL penicillin, and 100 μg/mL streptomycin, at 37°C in 5% CO_2_ atmosphere. After 24 h of seeding, cultures were washed with PBS, fresh medium was added, and the cells were treated with indicated doses of arsenicals and all-trans retinoic acid for indicated time.

### HPLC-ICP MS analysis

A polymer-based gel filtration column (Shodex Asahipak GS-220 HQ, 300 mm 7.6 mm i.d., Showa Denko, Tokyo) with an exclusion limit of 3000Da was used to separate unbound arsenic species from protein-bound arsenicals. A 20 μL aliquot of a sample solution was applied to the column, and then the column was eluted with 50 mM ammonium acetate buffers (pH6.5/7.0), at a flow rate of 0.6 mL/min or 0.8 ml/min, respectively. Arsenic species and concentrations were monitored with an Agilent 7500ce ICP-MS (Agilent Technologies, Tokyo, Japan). Arsenic (As) and Zinc (Zn) were monitored at *m*/*z*75 and 66 (respectively). Arsenic concentration was determined by ICP MS after wet-digestion with concentrated nitric acid (HNO_3_) and 30% H_2_O_2_ (v/*v* = 1/1) at 135°C for 2 days.

### Antibodies

Primary antibodies RARα, PRAM-1, rabbit anti-Poly (ADP-ribose) polymerase (PARP) polyclonal antibody, Cyt c, JNK, P-JNK, P-PERK, eif2α, rabbit anti-human PML, anti-human SUMO-1, Ub Antibody were purchased from Santa Cruz Biotechnology (CA, USA). Primary antibodies; Bax, BCL-2, β-actin, anti-cleaved caspase-3 antibody, caspase-3, Perk, P-ASK1, ASK, P-eif2α, GAPDH were purchased from Cell Signaling Technology (Danvers, MA). Anti-FLAG mouse monoclonal antibody was purchased from Sigma.

### MTT assay for cell proliferation

NB4 cells were seeded at a density of 2 × 10^4^ cells/100 μL/well in 96-well microtiter plates (Promega Corporation). Twenty-four hours post-seeding, the cultures were washed twice with PBS and then exposed to various concentrations of arsenicals. Then, 20 μL of an MTT solution was added to each well, and the plates were incubated for an additional 3 h at 37°C. Afterward, cell cultures were washed with PBS, and DMSO was added to each well. Cell proliferation was measured as absorbance at 570 nm with a microplate reader.

### Assay for CD11b and apoptosis

For CD11b determination, arsenicals (or ATRA)-treated NB4 cells were washed twice with PBS before incubation with FITC-conjugated anti-CD11b antibody, and then analyzed on flow cytometer (Beckmancoulter). For apoptosis, cells were stained with AnnexinV-FITC and PI for analysis of cellular apoptosis.

### Western blot analysis

NB4 cells were washed twice with D-hanks solution, followed by the lysis of cell pellets using RIPA lysis buffer (50 mM Tris-HCl, pH 7.5, 150 mM NaCl, 1% NP-40, 0.5% sodium deoxycholate, 0.1% SDS, 0.2 mM PMSF, and a complete mini protease inhibitor tablet) with 8M urea. Whole cell lysates were incubated on ice for 30 min and centrifuged for 30 min at 13,000*g* at 4°C to obtain the supernatant for western blot analysis. Notably, HEK 293T and HeLa cells were lysed in RIPA buffer without 8 M urea, and then incubated on ice and centrifuged as mentioned above to obtain the supernatant (S) and pellet (P) fractions. The supernatant was transferred and the pellet was washed with PBS, lysed in SDS buffer (1 × TBS, 10% glycerol, 0.015% EDTA, 50 mM DTT, and 2% SDS), boiled for 10 min at 95°C. Twenty-five microgram of each protein sample was resolved by 10~12% SDS-PAGE and electroblotted onto nitrocellulose membranes. The membranes were blocked with non-fat milk and incubated overnight with different primary antibodies at 4°C, followed by incubation with HRP-linked secondary antibodies for 1 h at room temperature and then the proteins were visualized by enhanced chemiluminescence.

### Immunoprecipitation assay

Immunoprecipitation was performed as described by Zhang et al. [18]. Briefly, HEK293T cells were transfected with *Flag-PML* using Lipofectamine 2000 (Invitrogen). After 24 h of post-transfection, the cells were treated with arsenicals and then lysed in IP buffer in an ice bath, followed by centrifugation at 14000*g* to obtain the supernatant. In addition, appropriate specific antibody, normal IgG and proteinA/G-beads were added to supernatant and incubated at 4°C for overnight. Then, removing the supernatant after centrifugation, the SDS loading buffer was added to the pellets and boiled for 10 min at 95°C. The samples were further centrifuged to obtain the supernatant for western blot analysis.

### Immunofluorescence microscopy

Arsenic-treated NB4 cells were transferred onto glass slides using a Shandon cytospin (Runcorn, UK), while *FLAG-PML (or FLAG-PML-RARα)* transfected HEK293T cells and HeLa cells were cultured on Chamber slides and then exposed to arsenicals. After washing with PBS twice, the slides were fixed in 4% paraformaldehyde and permeabilized with 0.1% Triton X-100, blocked with PBST with 10% fetal bovine serum. Further, the cells were then blocked with 1% BSA in PBS, followed by incubation with primary antibodies overnight at 4°C. Later they were tagged with fluorescent secondary antibodies for 2 h. Slides were mounted using Vectashield mounting liquid (Vector Labs) and sealed, stored in dark at 4°C. The cells were examined under a Zeiss (Göttingen, Germany) 510 confocal microscope.

### Statistical analysis

Each proliferation value represents the mean ± S.D. from four determinations, and IC_50_ values were calculated from the log-log plot between the percentages of viable cells. Subsequently, each experiment was performed at least three times. Statistical analysis of data was carried out using a one-way ANOVA followed by Holm-Sidak pairwise multiple comparison test (Sigmaplot, Systat Software Inc), and a probability value of less than 0.05 (*p < 0.05) was accepted as a significant difference.

## SUPPLEMENTARY DATA FIGURES



## Abbreviations

ATRA: all-trans retinoic acid
AS3MT: arsenic (+3 oxidation state) methyltransferase
As_2_O_3_: arsenic trioxide
iAs^III^: arsenite
iAs^V^: arsenate
APL: acute promyelocytic leukemia
Cyt c: cytochrome c
DMA^III^: dimethylarsinous acid
DMA^V^: dimethylarsinic acid
MMA^III^: monomethylarsonous acid
MMA^V^: monomethylarsonic acid
mPTP: mitochondrial permeability transition pore
HPLC: high performance liquid chromatography
ICP MS: inductively coupled argon plasma mass spectrometry
PML-RARα: Promyelocytic leukemia-Retinoic acid receptor
PML-NBs: PML-nuclear bodies
