# Supplementary material for: Methylated arsenic metabolites bind to PML protein but do not induce cellular differentiation and PML-RARα protein degradation
Source: Oncotarget. 2015 Jul 15;6(28):25646–59. doi: 10.18632/oncotarget.4662 (PMC4694856; doi:10.18632/oncotarget.4662)
Supplement: Supplementary file 1 [file oncotarget-06-25646-s001.pdf]

MATERIALS AND METHODS

Antibodies

The following antibodies were used in the current study: PRAM-1 (Cat No.sc-98879), Caspase-3 (Cat No.sc-7272), PARP-1/2 (Cat No.sc-7150), SUMO-2/3 (Cat No.sc-26969), Ub (Cat No.sc-8071), cytochrome C (Cat No.sc-13156), p-PERK (Cat No.sc-32577), eIF2α(Cat No.sc-30882), JNK (Cat No.sc-571) antibodies were purchased from Santa Cruz; GAPDH (Cat No.5174), β-actin (Cat No.3700s), Cleaved Caspase-3 (Cat No.9664s), COX IV (Cat No.11967s), CHOP (Cat No.5554s), p-JNK (Cat No.9251s), SUMO-1 (Cat No.4930s), Bax (Cat No.2772s), Bcl-2 (Cat No.2875s), PERK (Cat No.5683s), ATF4 (Cat No.11815s), ASK1 (Cat No.8662s), p-ASK1 (Cat No.3764s), p-eIF2α (Cat No.3597s) antibodies were purchased from Cell Signaling Technology; CD11b (Cat No.553311/557396) antibody was purchased from BD; RARα (Cat No.ab28767) antibody was purchased from Abcam; Flag (Cat No.F1804) antibody was purchased from Sigma.

Preparation of monomethylarsonous acid (MMA^{III}) and Dimethylarsinous Acid (DMA^{III})

MMA^{III} and DMA^{III} were prepared by reducing monomethylarsonic acid (MMA^{V}) and dimethylarsinic acid (DMA^{V}) respectively with 5 molar equivalents of L-cysteine in distilled water at 90°C for 1 h. The trivalent forms were confirmed by comparison to their iodide forms using gel filtration HPLC − ICP MS.

Mitochondrial membrane potential assay

The changes in mitochondrial membrane potential ($\Delta\Psi_m$) were monitored using 5, 5′, 6, 6′-tetrachloro-1, 1′, 3, 3′-tetraethylbenzimidazolcarbocyanine iodide dye (JC-1). Arsenic-exposed NB4 cells were stained with JC-1 (10 µg/mL) and kept in dark at 37°C for 15 min. Later cells were washed twice with PBS and then immediately analyzed by fluorescence microscopy.

Isolation of pure mitochondria from rat liver

All experiments on animal were carried out according to the "Principles of Laboratory Animal Care" (NIH version, revised 1996) and the Guidelines of the Animal Investigation Committee, School of Medicine, Zhejiang University, China (Permit No: ZJU2010101033).

Six week old male SD rats were purchased from National Rodent Laboratory Animal, Resource, Shanghai, China. The rats were housed in a humidity-controlled room, maintained at 22~25°C with a 12 h light dark cycle. The animals were fed commercial diet and tap water was provided. Following a one-week acclimatization period, rats at 7 weeks of age (body weight, 180–220 g) were used for experiments. Whole liver perfusion was performed following the method developed in our laboratory. Briefly, rats under sodium pentobarbital anesthesia were dissected to expose the heart, and then 0.2 mL of heparin was injected into the left ventricle. The remaining blood was perfused through the portal vein by a roller pump with phosphate buffer saline (PBS) at a flow rate of 6 mL/min. Blood-free liver was minced in ice-cold homogenization buffer A (230 mM Mannitol, 70 mM sucrose, 10 mM Tris-HCl, EDTA-2Na and 0. 5% bovine serum albumin (BSA), pH 7.4) by using a Dounce homogenizer to make 20% (w/v) liver homogenates. The homogenates were then kept on ice for 5 min to remove unbroken cells and connective tissues. After removing the unbroken cells, the supernatant was centrifuged twice at 700 g for 10 min at 4°C to obtain the cellular nuclear fraction and supernatant fraction. Mitochondria were isolated by subjecting the supernatant to centrifugation at 12, 000 g for 10 min at 4°C to obtain pellet. The pellet was washed twice and re-suspended in the isolation buffer B (230 mM Mannitol, 70 mM sucrose, 10 mM Tris-HCl, 1 mM EDTA· 2Na) with a protein concentration of 0.5 mg protein/mL. Isolated mitochondrial activity was determined by cytochrome c oxidase (CCO) assay kit.

Preparation of recombinant PML-R

The cDNAs encoding residue 49–104 of PML Zinc finger reign ring domain (R) was amplified according to the published paper, and inserted into pET32a using restriction sites Kpn I and Xho I to construct pET32a PML-R. Moreover, prokaryotic pET32M PML-R was transformed into *E.coli* BL21 (DE3) cells. Cells were grown in M9 minimal media and induced by 100 µM IPTG and 20 µM $ZnCl_2$. The Trx tag of Trx-PML-R was removed by Enterokinase (EK) cleavage. PML-R was further purified by Superdex-75 column chromatography and confirmed by SDS-PAGE.

Removing unbound arsenic compounds in supernatants of cells

The supernatants (500 µL) were dialyzed two times for 6 h each in a Slide-A-Lyzer Dialysis Cassette against 500 mL of 50 mM ammonium acetate buffer (pH 7.4) at 4°C, and the arsenic concentration in the supernatants was determined by ICP MS after wet-digestion with a mixture of concentrated nitric acid and 30% H_2O_2 (v/*v* = 1:1) at 150°C for 2 days.

Incubation of recombinant PML-R with the three arsenic compounds

Recombinant PML-R protein was diluted in 10mM Tris-HNO_3 buffer (pH7.4, dissolved oxygen was purged by bubbling with99.999% nitrogen gas), and incubated with 3 μM of iAs^{III}, MMA^{III} and DMA^{III} for 30 min at 37°C. Later 20 μL of each sample was subjected to HPLC-ICP MS analysis on GS-220 gel filtration columns with 50 mM ammonium acetate (pH7.0) at the flow rate of 0.8 mL/min. Arsenic (As) was monitored at m/z75.

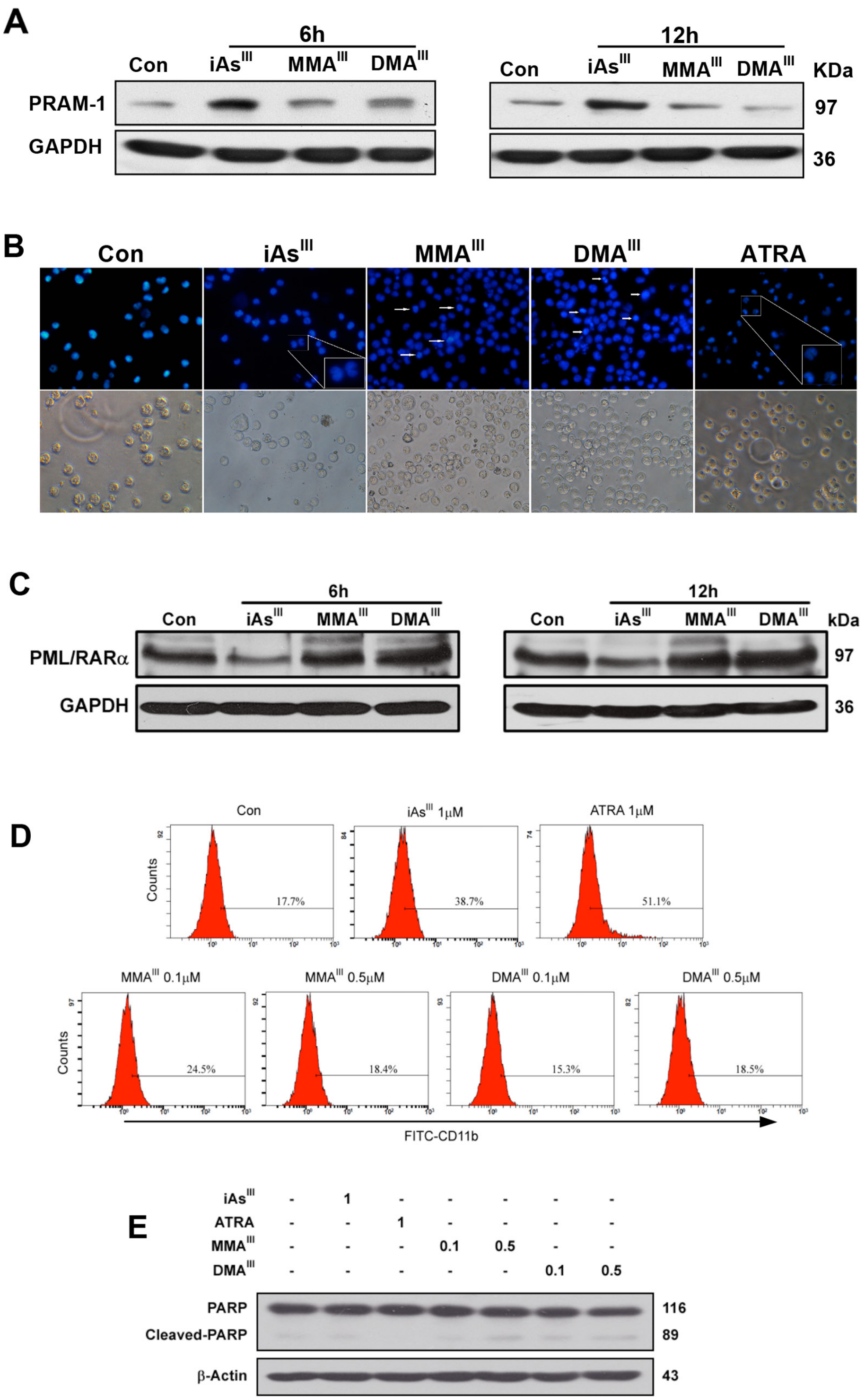

Supplementary Figure S1: Effects of iAs^{III} and its methylated intermediate metabolites on expression of PRAM-1 protein, PML-RARα fusion protein degradation and nuclear morphology in NB4 cells. PRAM-1 protein was determined in NB4 cells by exposure to three arsenic compounds (i.e., iAs^{III}, MMA^{III} and DMA^{III}) at 6 and 12 h **A.** In addition, cells were exposed to 1 μM of iAs^{III}, MMA^{III} and DMA^{III} for 24 h to determine the nuclear morphology **B.** Blue fluorescence indicates cell nucleus. Cells were imaged with fluorescence microscope. The arrows indicate cells with chromatin condensation. PML-RARα fusion protein was determined in NB4 cells after exposure to three arsenic compounds at 6 and 12 h by western blot **C.** Additionally, NB4 cells were exposed to methylated MMA^{III} and DMA^{III} at low doses (0.1 or 0.5 μM) for three days and then the CD11b was determined by flow cytometry **D.** PARP and cleaved PARP were determined by western blot **E.**

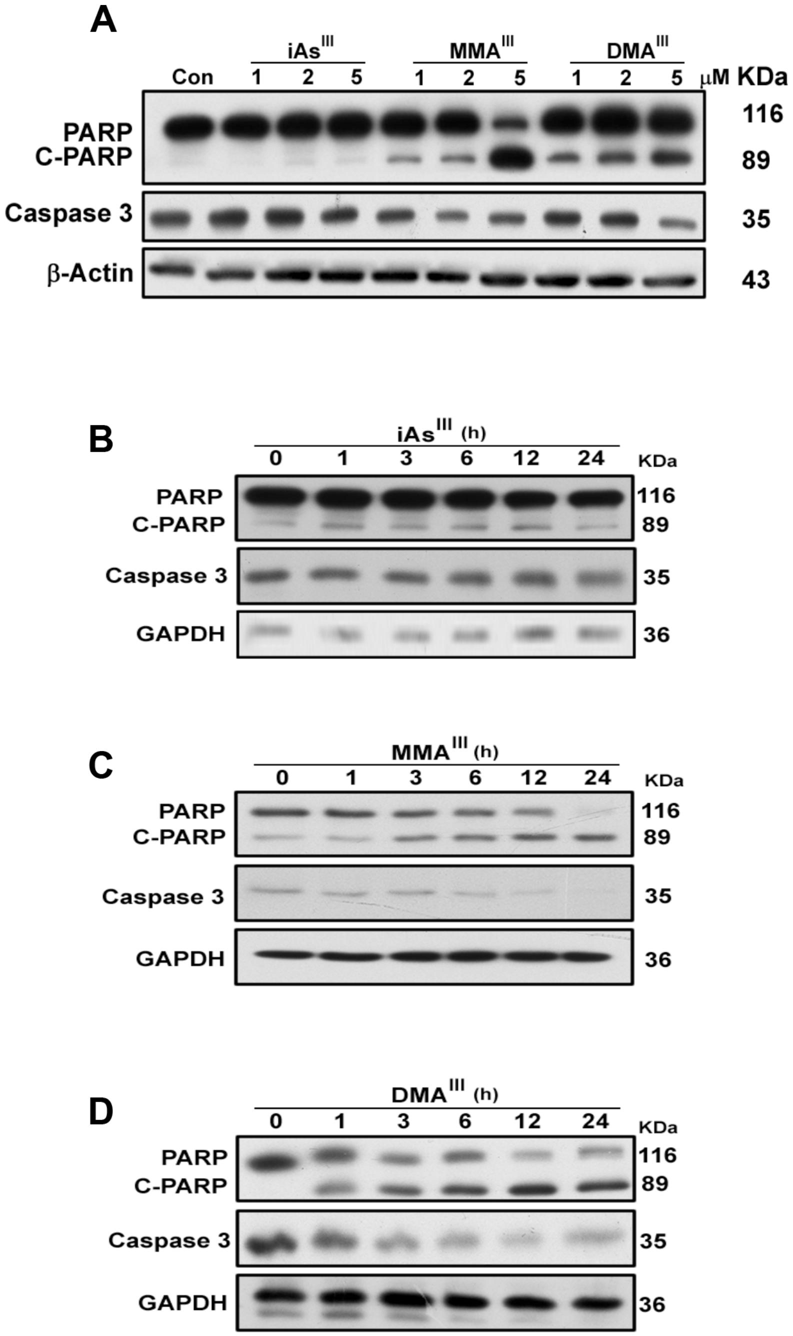

Supplementary Figure S2: Effect of arsenic compounds on induction of apoptosis in NB4 cells in a dose- and time-dependent manner. NB4 cells were exposed to 1, 2 and 5 μM of iAs^{III}, MMA^{III} and DMA^{III} for 6 h **A.** or for 1, 3, 6, 12 and 24 h with 1 μM of iAs^{III} **B.** MMA^{III} **C.** and DMA^{III} **D.** Changes in PARP, cleaved PARP and Caspase-3 proteins were determined by western blot.

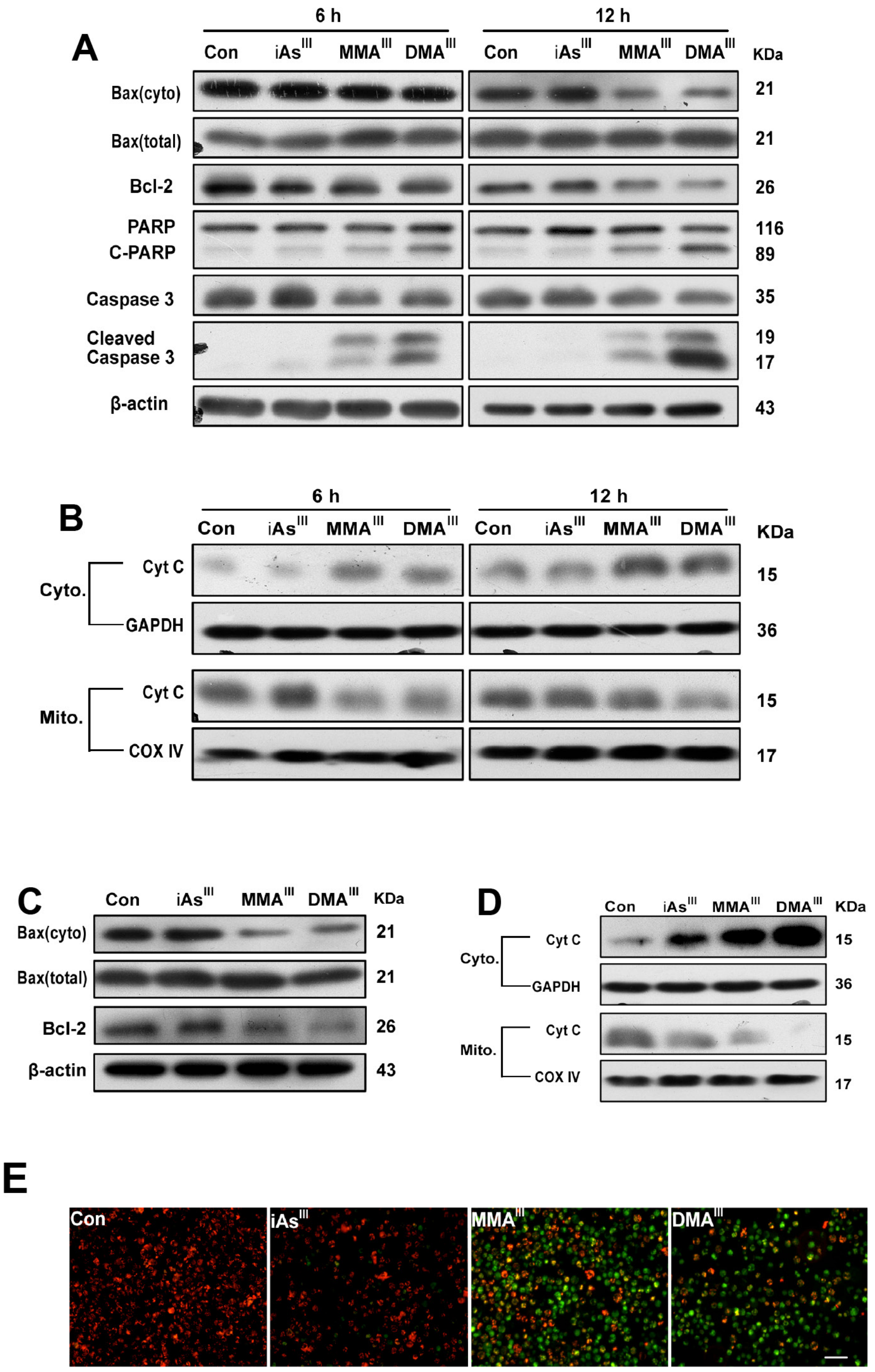

Supplementary Figure S3: Changes in apoptosis related proteins in NB4 cells after exposure to three arsenic compounds. NB4 cells were exposed to 1 μM of iAs^{III}, MMA^{III} and DMA^{III} for 6 and 12 h to determined apoptosis related proteins (e.g., caspase-3, cleaved-caspase-3, PARP-1, Bax, Bcl-2 and cleaved PARP-1) **A.** Likewise, changes in Cyt c in mitochondria (Mito) and cytoplasm (Cyto) were determined at 6 and 12 h in NB4 cells after exposure to three arsenic species **B.** Additionally, changes in Bax, Bcl-2 **C.** and Cyt c **D.** in cytoplasm or mitochondria of NB4 cells were further determined at 24 h by western blot. The mitochondrial membrane potential ($\Delta\Psi_m$) was determined in NB4 cells by using the dual-fluorescent dye JC-1 6 h after exposure to arsenicals **E.**

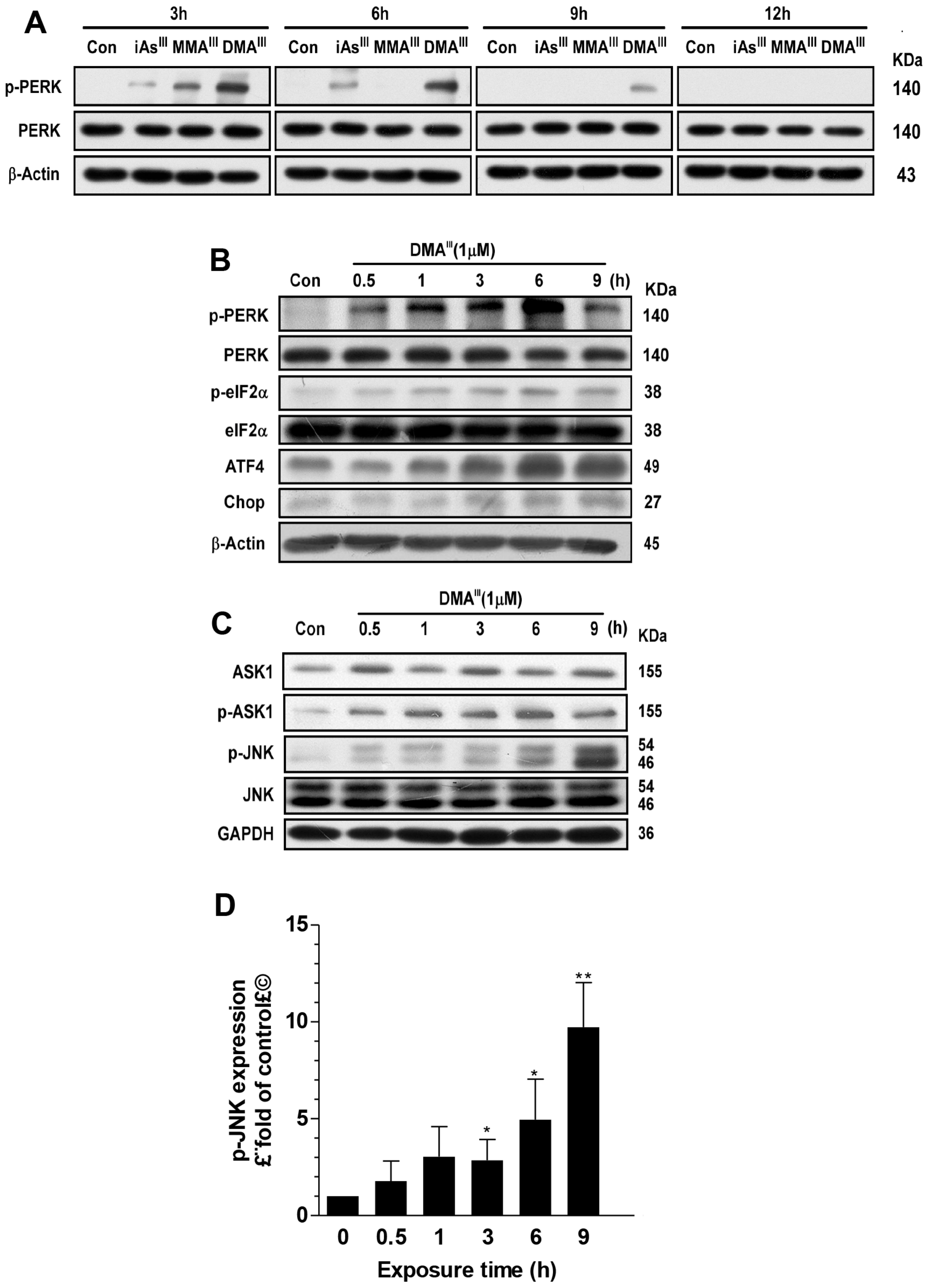

Supplementary Figure S4: Induction of ER-stress in NB4 cells after exposure to three arsenic compounds. NB4 cells were treated with 1 μM of iAs^{III}, MMA^{III} and DMA^{III} for 3, 6, 9 and 12 h to examine the changes in phospho-PERK protein **A.** followed by determination of the expression of phospho-PERK, phospho-eIF2a, ATF4 and CHOP **B.** or phospho-ASK-1 and JNK in cells after exposure to DMA^{III} (1 μM) **C.** Analyses of band intensity on films are presented as the relative ratio of p-JNK to actin **D.** Asterisks indicate significance difference from the corresponding compared group at $*p < 0.05$.

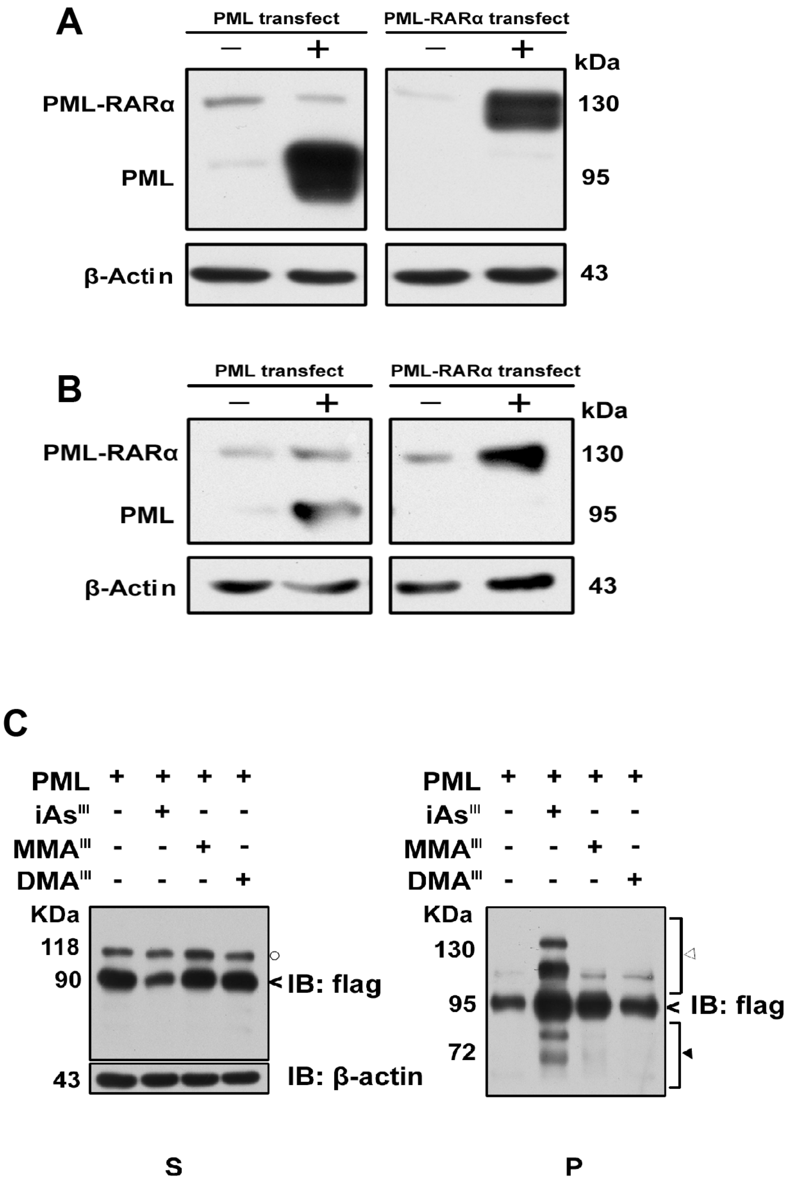

Supplementary Figure S5: Effect of three arsenic compounds on degradation PML-RARα fusion protein or modification of PML proteins in HeLa cells. HEK293T cells **A.** or HeLa cells **B.** were transiently transfected with *PML* and *PML-RARα* genes for 24 h, and protein expression efficacy was evaluated by western blot. On the other hand, *Flag-PML* expressed HEK293T cells were exposed to 4 µM of iAs^{III}, MMA^{III} and DMA^{III} for 1 h, and the whole cell lysate in RIPA buffer was centrifuged to obtain supernatant (S) and pellet (P) fractions for the determination of PML proteins (<), modified PML-proteins (◀) and degraded PML-protein (◀) by Western blot analysis **B.** PML protein was immunoprecipitated from HEK293T cells extract expressing FLAG-PML using anti-FLAG M2 antibody after exposure to arsenic species at different time points **C.**

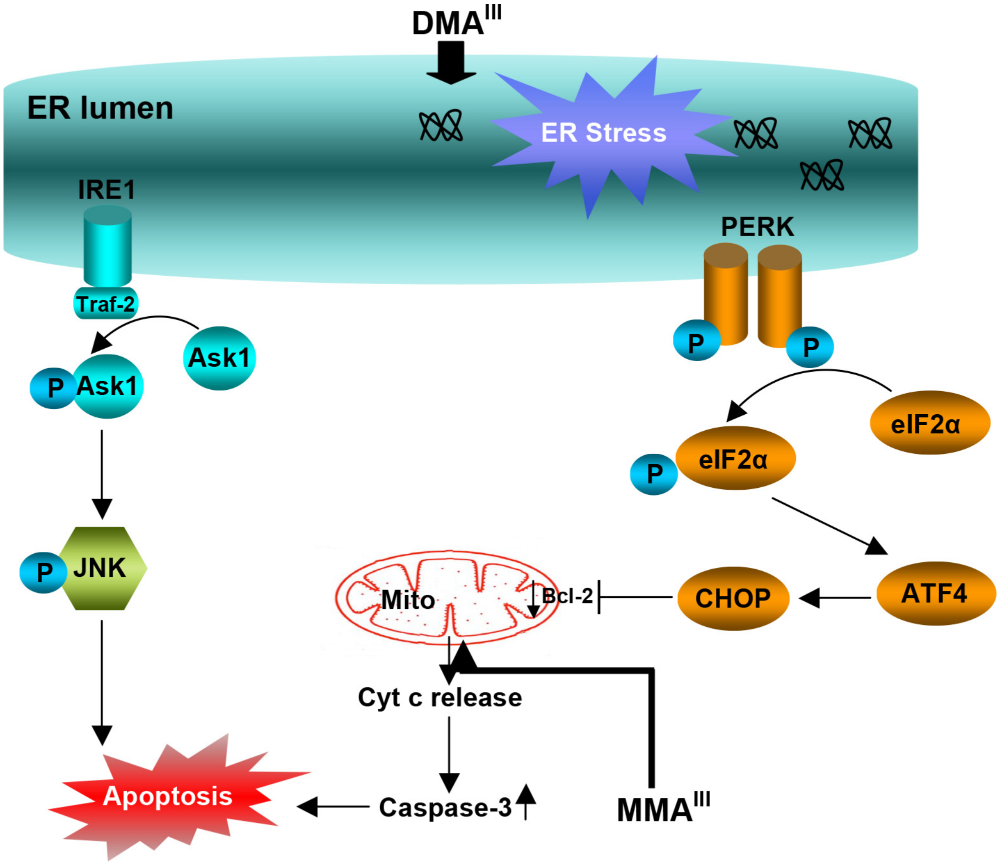

Supplementary Figure S6: Proposed mechanisms underlying the methylated trivalent arsenic species-induced cell death in NB4 Cells.
